# Multi-parametric assessment of left ventricular hypertrophy using late gadolinium enhancement, T1 mapping and strain-encoded cardiovascular magnetic resonance

**DOI:** 10.1186/s12968-021-00775-8

**Published:** 2021-07-12

**Authors:** Sorin Giusca, Henning Steen, Moritz Montenbruck, Amit R. Patel, Burkert Pieske, Jennifer Erley, Sebastian Kelle, Grigorios Korosoglou

**Affiliations:** 1 Departments of Cardiology, Vascular Medicine and Pneumology, GRN Hospital Weinheim, Roentgenstrasse 1, 69469 Weinheim, Germany; 2 Department of Cardiology, Marien Hospital Hamburg, Hamburg, Germany; 3 Department of Medicine, University of Chicago, Illinois, USA; 4 Department of Internal Medicine, Cardiology German Heart Center Berlin, Berlin, Germany; 5 DZHK (German Center for Cardiovascular Research), Partner Site Berlin, Berlin, Germany

**Keywords:** Fast strain-encoded CMR (fast-SENC), Myocardial hypertrophy, Hypertrophic cardiomyopathy, Hypertensive heart disease, Athletes’ heart, Cardiac amyloidosis, T1 mapping, Atypical late gadolinium enhancement

## Abstract

**Aim:**

To evaluate the ability of single heartbeat fast-strain encoded (SENC) cardiovascular magnetic resonance (CMR) derived myocardial strain to discriminate between different forms of left ventricular (LV) hypertrophy (LVH).

**Methods:**

314 patients (228 with hypertensive heart disease (HHD), 45 with hypertrophic cardiomyopathy (HCM), 41 with amyloidosis, 22 competitive athletes, and 33 healthy controls) were systematically analysed. LV ejection fraction (LVEF), LV mass index and interventricular septal (IVS) thickness, T1 mapping and atypical late gadolinium enhancement (LGE) were assessed. In addition, the percentage of LV myocardial segments with strain ≤ − 17% (%normal myocardium) was determined.

**Results:**

Patients with amyloidosis and HCM exhibited the highest IVS thickness (17.4 ± 3.3 mm and 17.4 ± 6 mm, respectively, p < 0.05 vs. all other groups), whereas patients with amyloidosis showed the highest LV mass index (95.1 ± 20.1 g/m^2^, p < 0.05 vs all others) and lower LVEF compared to controls (50.5 ± 9.8% vs 59.2 ± 5.5%, p < 0.05). Analysing subjects with mild to moderate hypertrophy (IVS 11–15 mm), %normal myocardium exhibited excellent and high precision, respectively for the differentiation between athletes vs. HCM (sensitivity and specificity = 100%, Area under the curve; AUC_%normalmyocardium_ = 1.0, 95%CI = 0.85–1.0) and athletes vs. HHD (sensitivity = 83%, specificity = 75%, AUC_%normalmyocardium_ = 0.85, 95%CI = 0.78–0.90). Combining %normal myocardial strain with atypical LGE provided high accuracy also for the differentiation of HHD vs. HCM (sensitivity = 82%, specificity = 100%, AUC_combination_ = 0.92, 95%CI = 0.88–0.95) and HCM vs. amyloidosis (sensitivity = 83%, specificity = 100%, AUC_combination_ = 0.83, 95%CI = 0.60–0.96).

**Conclusion:**

Fast-SENC derived myocardial strain is a valuable tool for differentiating between athletes vs. HCM and athletes vs. HHD. Combining strain and LGE data is useful for differentiating between HHD vs. HCM and HCM vs. cardiac amyloidosis.

**Supplementary Information:**

The online version contains supplementary material available at 10.1186/s12968-021-00775-8.

## Background

Left ventricular (LV) hypertrophy (LVH) can be part of an adaptation process in athletes; due to increased afterload in patients with hypertensive heart disease (HHD); or an expression of myocyte hypertrophy and disarray in hypertrophic cardiomyopathy (HCM). In addition, systemic diseases affecting the heart, such as amyloidosis, also result in increased LV wall thickness (WT) [1]. Identifying the underlying disease in such patients is of paramount importance, because of substantial differences in therapeutic options.

Echocardiography provides accurate measurement of the LV mass and WT and, if required, myocardial strain, which were shown to aid the differential diagnosis of patients with LVH [2]. Despite its wide availability, echocardiography is dependent on the patient’s acoustic window and the operator’s skills, exhibiting observer variabilities [3]. Cardiovascular magnetic resonance (CMR) on the other hand, allows for a multiparametric approach in the evaluation of patients with LVH, providing information on cardiac morphology, function, as well as tissue characterisation (T1 mapping) and the late gadolinium enhancement (LGE), all in one examination [4]. In addition, advanced tagged sequences, such as fast strain-encoded CMR (fast-SENC), enable quantification of strain at free breathing and with high reproducibility [5]. The incremental value of this sequence for the diagnosis and risk stratification of patients with different cardiac diseases has been recently reported [6].

Herein we sought to evaluate to ability of fast-SENC derived LV strain to distinguish between different forms of LVH in competitive athletes, HHD, HCM and  cardiac amyloidosis. The ability of %normal myocardium by fast-SENC was compared to that of LV ejection fraction (LVEF), T1, LV mass index and to LGE.

## Methods

### Study population

The study was performed between September 2017 and February 2019 at the Marien Hospital Hamburg, Hamburg, Germany and German Heart Center Berlin, Berlin, Germany). During this period 1566 CMR scans were performed. Patients were selected with a clinical indication for the CMR examination that was related to (1) further evaluation of LVH diagnosed by echocardiography (2) evaluation of an underlying cause for symptoms of heart failure (3) evaluation of the presence and extent of scar tissue in patients with suspected or known history of cardiomyopathy. A total of 314 met criteria. In addition, 22 competitive athletes and 33 healthy subjects were included. All healthy subjects were free of any history of medical conditions, were on no medication at the time of the CMR, had a normal electrocardiogram (ECG), and a normal physical examination.

### Definitions of the underlying clinical entities

LVH was defined in patients with HHD, HCM, cardiac amyloidosis and in athletes according to current recommendations [7–9]. Thus, LVH was defined as an end diastolic WT > 12 mm in any LV segment [10]. In concordance to current recommendations, HHD was defined as the presence of myocardial hypertrophy in patients with known arterial hypertension and without any other cause for LVH. HCM was defined as an end-diastolic WT > 15 mm (or > 13 mm in first degree relatives of patients with HCM) [8], whereas the diagnosis of cardiac amyloidosis was based on the Congo red staining when available or using standard non-invasive diagnostic criteria [9]. The 22 competitive athletes (marathon or triathlon runners, bikers, or football players) trained for at least 3 years and for at least 5 h/week with intensive aerobic and anaerobic exercise.

### Conventional CMR protocol and data analysis

All CMR examinations and protocols were performed by clinical indication and conformed with the declaration of Helsinki. The use of patient data for research purposes was approved by the local ethics committees and all patients gave written informed consent. For conventional and LGE acquisitions, a standard CMR protocol was used, as described elsewhere [11]. The analysis of conventional CMR data was performed on commercially available workstation (cvi42, Circle Cardiovascular Imaging Inc., Calgary, Alberta, Canada). After the overview of thorax and scout images are acquired, the cine images are obtained using a balanced steady state free precession protocol in 3 long axes (2 chamber view, 4 chamber view and 3 chamber view) as well as a stack of short axis covering the entire LV. LVEF and volumes were calculated by standard measures. In addition, myocardial contraction fraction (MCF), the dimensionless ratio of LV stroke volume to LV myocardial volume was calculated. LV myocardial volume was calculated by dividing LV mass by 1.05.

T1 mapping acquisitions were performed using a standard modified Look-Locker inversion recovery (MOLLI) 5 s (3 s) 3 s T1 native sequence in standard mid-ventricular short axis views. Phase sensitive LGE images are acquired 10 min after intravenous injection of 0.1 mmol/kg of gadoterate meglumine. For the LGE analysis, three long axis and multiple short axes covering the entire LV were acquired. Of note, LV mass was measured without including the papillary muscles. Care was taken when measuring septal WT to exclude trabeculations arising from the right ventricle (RV) [12].

### Analysis of atypical LGE score and patterns

Each myocardial segment was analysed for the presence or absence of atypical LGE. In addition, different atypical LGE patterns were considered, including (1) diffuse or patchy LGE, (2) focal intramyocardial LGE and (3) focal epicardial LGE. For the calculation of a semiquantitative LGE score, segments with atypical LGE were summed up in each patient, and the resultant number was then divided by the total number of segments (n = 17). For the determination of the LGE pattern by patients, the most frequent pattern of LGE number in the affected segments was considered. Thus, if a patient for example with HCM had 2 segments with diffuse LGE and 3 additional segments with focal intramyocardial LGE pattern, the latter was considered for analysis, regarding the atypical LGE pattern on a patient-by-patient analysis. Patients with the same number of segments of 2 or 3 different LGE patterns were excluded from analysis.

### Single-Beat fast-SENC acquisitions (the fast-SENC pulse sequence)

The protocol for this sequence is described elsewhere [5, 13]. By combining spiral imaging and interleaved tuning, a cine acquisition can be acquired in a short interval corresponding to a single heartbeat. With fast-SENC, the modulation gradient is applied perpendicular to the slice-selection direction. Consequently, longitudinal strain is extracted from short axis and circumferential strain from long axis images. With our protocol, three short axis (basal, mid, and apical) and three long axis (3 chamber, 4 chamber and 2 chamber) acquisitions are performed. Typical imaging parameters are as follows: field-of-view = 256 × 256 mm^2^, slice thickness = 10 mm, voxel size = 4 × 4 × 10 mm^3^, reconstructed resolution = 1 × 1 × 10 mm^3^, single-shot spiral readout with flip angle = 30°, effective echo time (TE) = 0.7 ms, repetition time (TR) = 12 ms, temporal resolution = 36 ms, typical number of acquired heart phases = 22, spectrally selective fat suppression (SPIR), total acquisition time per slice < 1 s [5, 5]. The three short axes and three long axes acquired using the fast-SENC protocol and imported into a dedicated software (MyoStrain software, Myocardial Solutions, Inc., Morrisville, North Carolina, USA). The endocardial and epicardial borders were manually traced at the end-systolic frame and the tracking was checked, and when necessary, corrected throughout the entire cardiac cycle.

### Calculation of myocardial strain and of %normal myocardium by fast-SENC

The fast-SENC method uses out-of-plane phase encoding gradients along the slice-selected direction and thus longitudinal strain was extracted from the three short axes acquisitions using a 16-segment model, whereas circumferential strain was measured from three long axes views using a 21-segment model [14]. The global longitudinal strain (GLS) and global circumferential strain (GCS) values for myocardial strain were calculated as an average of the 16 and 21 segments, respectively.

In concordance with previous studies, we considered a value for either longitudinal or circumferential strain in any segment ≤ −17% as normal [15, 16]. We then measured the percentage of normal myocardium in each patient as the ratio between the total number of segments expressing normal myocardium, i.e. longitudinal strain ≤ −17% (out of n = 16) and circumferential ≤ −17% (out of n = 21) and then dividing this number by the total number of segments analysed (37 segments in total), as follows [17]:

$$\% normal\,myocardium = \frac{Segments\,with\,circumferential\,\& \,longitudinal\,strain \le - 17\% }{{37}}$$

The number of segments with diagnostic image quality, enabling the assessment of myocardial strain by fast-SENC was assessed in 80 randomly selected patients (n = 2960 segments).

In addition, we calculated a relative regional ratio of the average of the apical segmental strain divided by the sum of the average basal and mid-ventricular segmental strain, to differentiate between amyloidosis and HCM, as previously described [18].

To avoid confusion and in keeping with most of the literature on the subject, the strain parameters will be interpreted in their absolute values (i.e., more “negative” strain meaning better strain).

### Statistical analysis

Values are presented as mean ± standard deviation (SD) when normally distributed or as median and inter-quartile range for intervals without a normal distribution. A paired t-test was used to compare two groups of normally distributed values. The ANOVA test was used for comparing three or more normally distributed groups with the Scheffé test for post-hoc analysis [19]. The analysis was repeated using ANCOVA, to test for the influence of potential confounders such as age and sex. The Mann–Whitney test was used to compare ordinal variables and Fisher test to compare nominal variables. A Receiver Operator Characteristics (ROC) analysis was used to identify the best parameter that differentiates between different forms of LVH. Comparison of the Areas Under the Curve (AUC) of paired data ROC curves was performed using the DeLong method [20]. A Pearson correlation test was employed to test the relation between strain parameters and clinical and conventional CMR data. Cox proportional-hazards models were assessed including the following hierarchical steps: (1) atypical LGE score and (2) %normal myocardium. Furthermore, a clustered based approach with multivariable modeling was used to define a prediction score composed of multiple parameters, best separating patients with different underlying clinical entities. In addition, our cohort was randomly spit to 2 equally large cohorts, including cohort A, as a test and cohort B, as a validation cohort, respectively. In cohort A we defined optimal cut-off values for the differentiation between the various pathologies. These threshold values were then applied in the validation cohort B. Inter- and intra-observer variabilities for strain values were assessed by repeated analysis of 40 randomly selected patients and were calculated as the ratio of the standard deviation to the mean. All p values < 0.05 were considered statistically significant.

## Results

Data were available for 369 individuals, including 41 pts with amyloidosis, 45 pts with HCM, 228 with HHD, 22 athletes and 33 healthy subjects. All individuals included in the study were Caucasian and all studies exhibited diagnostic image quality, enabling the assessment of myocardial strain. Thus, using fast-SENC, 0.39% (5 of 1280) segments were excluded from analysis for the assessment of longitudinal and 0.48% (8 of 1680) for the assessment of circumferential strain, respectively.

**Fig. 1:**
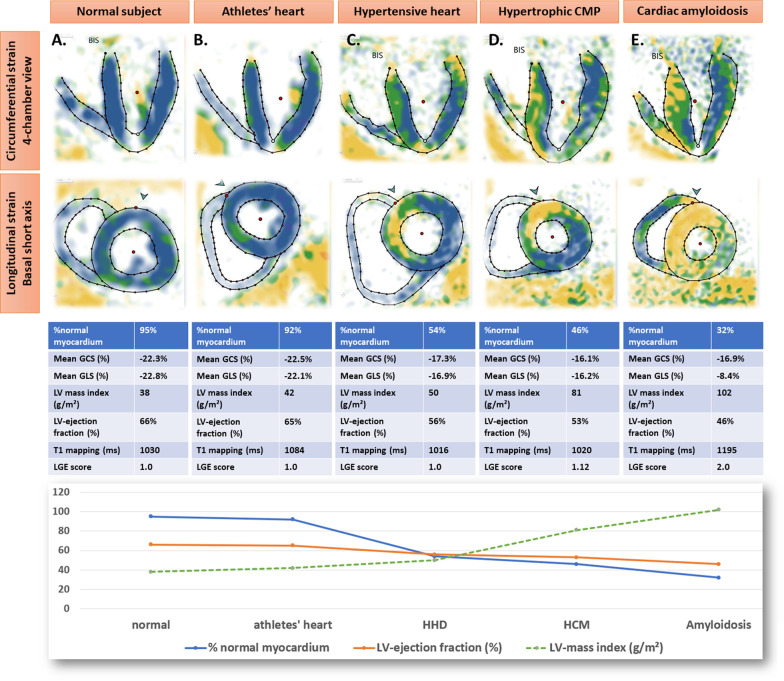
Representative cases of a healthy subject (**A**) and of left ventricular (LV) hypertrophy (LVH) in an athlete (**B**) and in patients with hypertensive heart disease (HHD) (**C**), hypertrophic cardiomyopathy (HCM) (**D**) and cardiac amyloidosis (**E**), respectively

Table 1 presents clinical, demographic and CMR data. Healthy subjects showed no LGE. Competitive athletes exhibited the lowest amount of LGE, followed by HHD and then by HCM, whereas patients with amyloidosis exhibited the highest amount of LGE. In addition, only focal LGE was found in athletes, whereas diffuse/patchy LGE was increasingly found in HHD and HCM. Patients with amyloidosis showed only diffuse LGE pattern. Differences were observed for several variables, including age, LVEF, strain, MCF, and LV volumes, which remained statistically significant after adjusting for age and sex. Figure 1 shows typical systolic fast-SENC images and strain, T1 and LGE score values.

**Table 1:** Demographic, clinical and CMR data from the studied cohorts

	Healthy subjects (33 pts)	Athletes (22 pts)	HHD (228 pts)	HCM (45 pts)	Amyloidosis (41 pts)	p values*
Age (years)	31.7 ± 10	37.1 ± 11	66.2 ± 11	56 ± 15	71.4 ± 11	< 0.001
Female sex	13 (39%)	7 (32%)	74 (32%)	17 (38%)	3 (7%)	0.01
Arterial hypertension	0 (0%)	0 (0%)	228 (100%)	30 (67%)	25 (61%)	< 0.001
Diabetes mellitus	0 (0%)	1 (4%)	53 (19%)	8 (18%)	5 (12%)	< 0.001
BMI (Kg/m^2^)	23.9 ± 5.6	22 ± 2.5	28.2 ± 4.8	27 ± 4.4	24.4 ± 2.5	< 0.001
BSA (m^2^)	1.8 ± 0.2	1.9 ± 0.2	2 ± 0.2	2 ± 0.2	1.9 ± 0.2	< 0.01
LVEDV (ml)	183.6 ± 46	206.2 ± 43	166.5 ± 41	171 ± 38	181.3 ± 42	0.001
LVEDV index (ml/m^2^)	89 ± 20	107.3 ± 16	82.3 ± 17	86 ± 17.1	94.1 ± 21	0.001
LVESV (ml)	60 ± 19	85.2 ± 28	66.4 ± 29	72.3 ± 54	85.6 ± 33	< 0.01
LVESV index (ml/m^2^)	32.4 ± 8	44 ± 11	32.5 ± 12	32.4 ± 11	44.2 ± 17	< 0.001
LV ejection fraction	59.2 ± 5	55 ± 7	55 ± 8.5	56 ± 10	50 ± 10	< 0.01
Stroke volume (ml)	109 ± 31	112 ± 24	91 ± 23	95 ± 29	91 ± 24	< 0.001
Stroke volume index (ml/m2)	51 ± 12	59 ± 11	45 ± 11	48 ± 12	47 ± 12	< 0.001
IVS (mm)	7.6 ± 1.7	9 ± 2	12 ± 1.8	17.4 ± 6	17.4 ± 3.3	< 0.001
Lateral wall (mm)	5.2 ± 1.5	6.8 ± 2.3	8 ± 2	8.7 ± 3	12.3 ± 3.5	< 0.001
LV mass (g)	108.2 ± 24	134.2 ± 30	130 ± 30	154 ± 50.6	184.1 ± 45	< 0.001
LV mass index (g/m^2^)	56.1 ± 11	69.5 ± 11	64.2 ± 13	76.8 ± 21	95 ± 21	< 0.001
MCF	1.07 ± 0.23	0.91 ± 0.21	0.75 ± 0.17	0.69 ± 0.21	0.55 ± 0.19	< 0.001
LV concentricity	0.69 ± 0.1	0.68 ± 0.12	0.84 ± 0.17	0.96 ± 0.25	1.14 ± 0.25	< 0.001
T1 (ms)	1052 ± 27	1041 ± 42	1054 ± 44	1079 ± 61	1175 ± 65	< 0.001
Atypical LGE present	0 (0%)	3 (14%)	81 (36%)	44 (98%)	37 (90%)	< 0.001
Diffuse LGE present	0 (0%)	0 (0%)	25 (11%)	21 (47%)	37 (90%)	< 0.01
Focal intramyocardial LGE	0 (0%)	2 (9%)	32 (14%)	21 (47%)	0 (0%)	< 0.01
Focal epicardial LGE	0 (0%)	1 (5%)	24 (11%)	2 (5%)	0 (0%)	< 0.05
Distribution of focal vs. diffuse LGE	N.A	1.0 ± 0	1.2 ± 0.4	1.5 ± 0.5	2.0 ± 0	< 0.001
Atypical LGE score	1.00 ± 0	1.03 ± 0.09	1.06 ± 0.09	1.25 ± 0.19	1.75 ± 0.39	< 0.001
GLS(%)	− 20.9 ± 1.2	− 20.2 ± 1.2	− 18.8 ± 2.1	− 14.7 ± 3.5	− 12.2 ± 3	< 0.001
GCS(%)	− 20.2 ± 1.6	− 19.9 ± 1.3	− 17.8 ± 1.8	− 16.3 ± 2.2	− 14.1 ± 2.6	< 0.001
%normal myocardium	0.85 ± 0.06	0.83 ± 0.06	0.66 ± 0.15	0.48 ± 0.15	0.27 ± 0.18	< 0.001

*BMI* body-mass-index, *BSA* body surface area, *LV* left ventricle, *EDV* end-diastolic-volume, *ESV* end-systolic-volume, *IVS* intraventricular septum, *LGE* late gadolinium enhancement, *GLS* global longitudinal strain, *GCS* global circumferential strain, *MCF* myocardial contraction fraction, *N.A.* not applicable

LV concentricity was calculated as a ratio of LV mass divided by LVEDV

*Statistical significance remained after adjustment for the co-variates “age” and “sex”

### Correlation between myocardial strain and other CMR parameters

Poor correlations were observed between LV longitudinal, LV circumferential strain and %normal myocardium with LVEF, whereas moderate correlations were depicted between %normal myocardium and LV mass, T1 values and atypical LGE score index (Additional file 1: Figure S1).

### Myocardial strain and other CMR parameters with different forms of LV hypertrophy

**Fig. 2:**
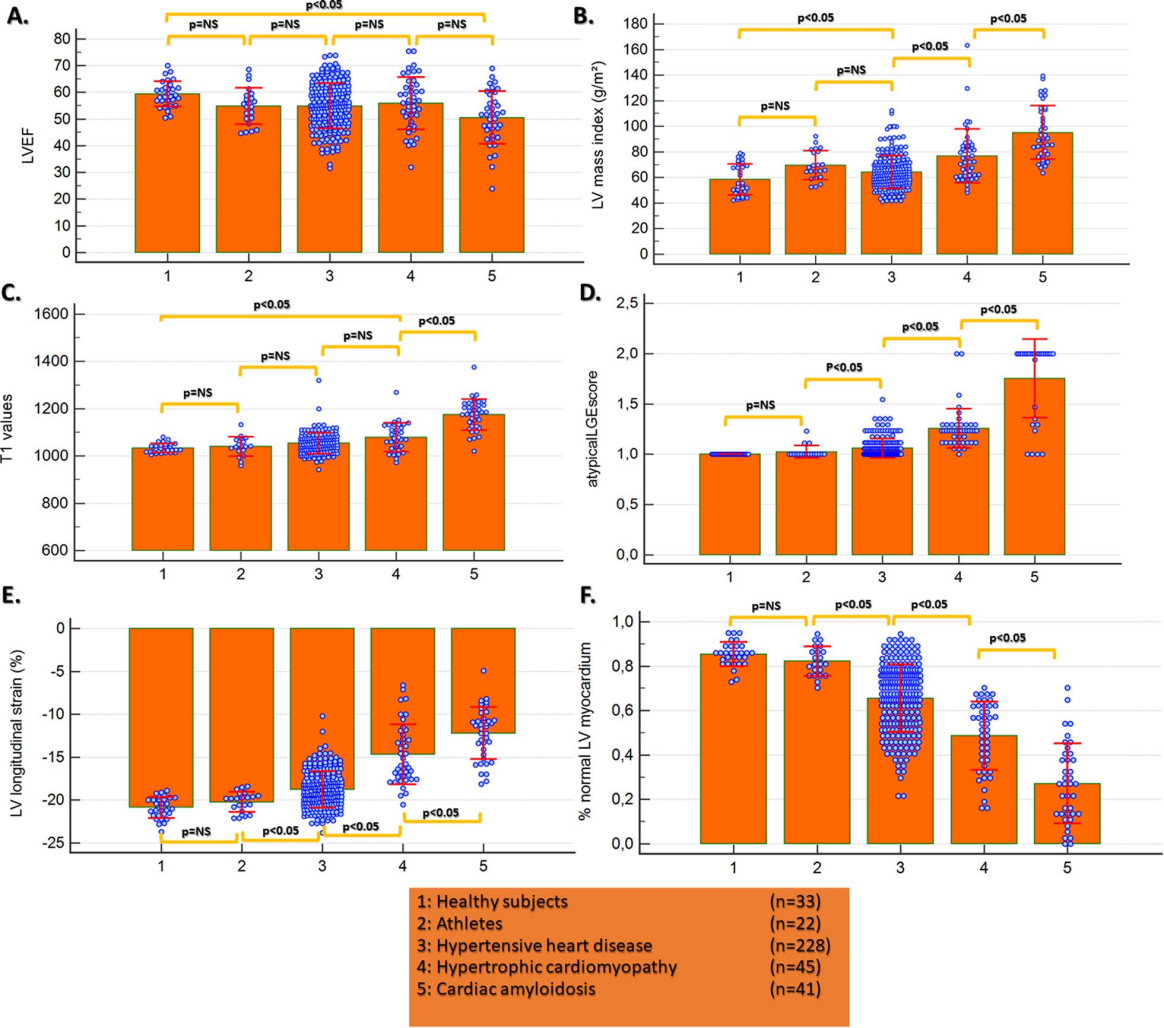
Apart from patients with cardiac amyloidosis, there was no difference in LV ejection fraction (LVEF) between the other groups (**A**). Patients with HCM and amyloidosis exhibited the highest LV mass index (**B**). Patients with cardiac amyloidosis exhibited the highest values for T1 followed by HCM patients (p < 0.05 vs. controls). There were no significant differences in T1 however, between athletes, healthy controls and HHD patients (**C**). Atypical late gadolinium enhancement (LGE) score was higher in HHD and HCM vs. controls and even higher in amyloidosis (**D**). GLS and %normal myocardium were similar between healthy subjects and athletes, whereas values significantly decreased with HHD and further decreased with HCM and amyloidosis (**E**, **F**)

Apart from patients with amyloidosis, who had lower LVEF (50 ± 10%, p < 0.05 vs. healthy subjects), there were no differences between the other groups. Patients with HCM and cardiac amyloidosis exhibited the highest LV mass index. Patients with cardiac amyloidosis exhibited the highest values for T1, followed by HCM patients (p < 0.05 vs. healthy controls). There were no significant differences in T1 however, between athletes, healthy controls and HHD patients. Atypical LGE score index was significantly higher in HHD and HCM and even higher with amyloidosis. GLS and %normal myocardium were similar between healthy subjects and athletes, decreased with HHD and further decreased with HCM and amyloidosis (Fig. 2).

### Differentiating between mild to moderate LV hypertrophy

**Fig. 3:**
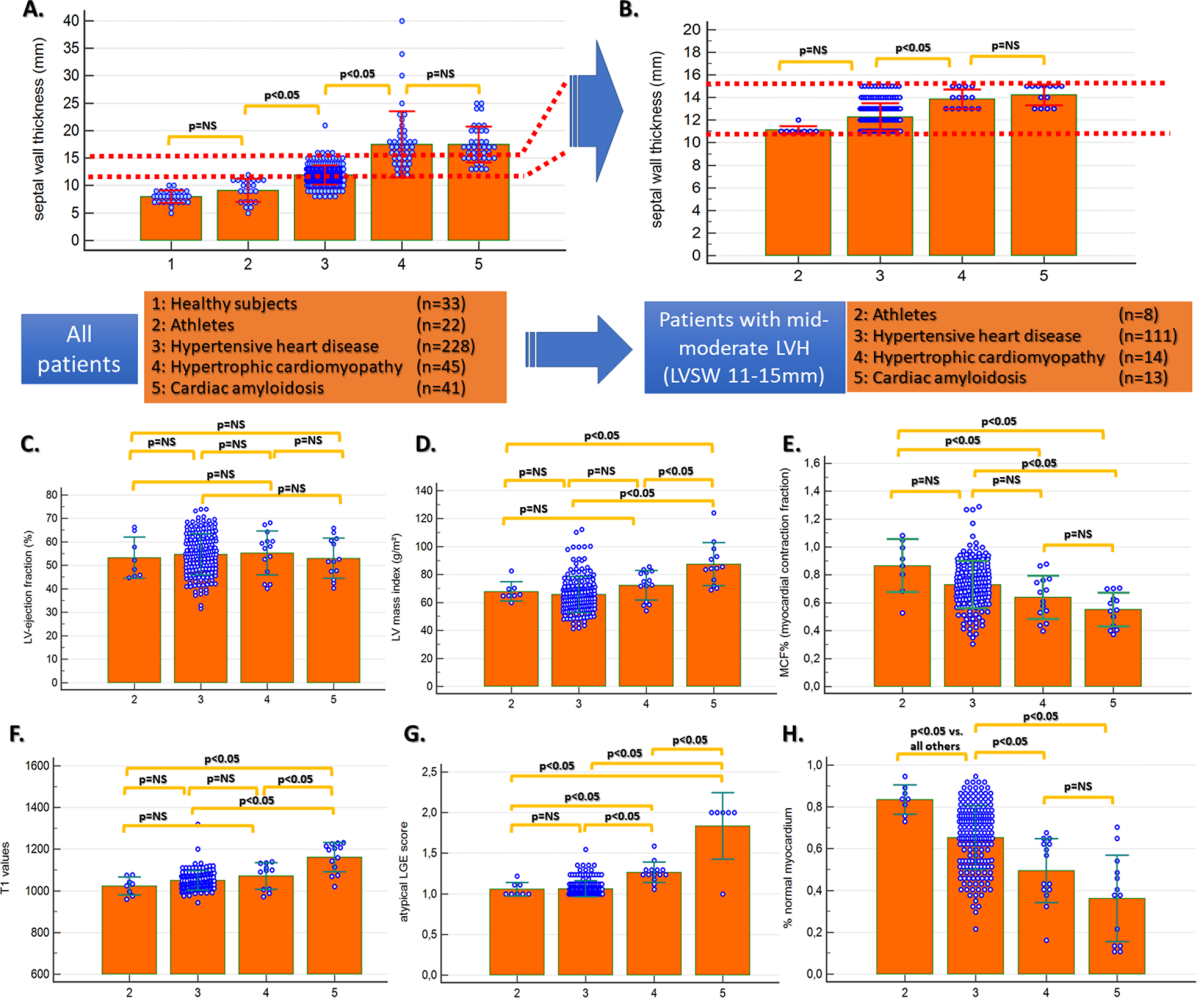
Due to substantial differences in septal wall thickness (**A**), subsection analysis was performed in patients with mild to moderate LV hypertrophy (IVS 11–15 mm, as shown by the red bars in **A**-**B**). LV ejection fraction was similar between the four subgroups (**C**), whereas only patients with cardiac amyloidosis exhibited a higher LV mass index and T1 values compared to all other groups (**D** and **F**). MCF was lower in patients with cardiac amyloidosis compared with athletes and HHD but like HCM (**E**). Atypical LGE differentiated between amyloidosis and all other hypertrophy forms, between athletes and HHD or HCM, but not between athletes and HHD (**G**). %normal myocardium differentiated between all hypertrophy forms, except between HCM and amyloidosis (**H**)

Subgroup analysis was conducted in patients with mild to moderate LVH with a septal thickness between 11 and 15 mm (red bars in Fig. 3a) in 8 athletes (range 11–12 mm), 177 pts with HHD (range 11–15 mm), 14 patients with HCM (range 13–15 mm) and 13 patients with amyloidosis (range 13–15 mm) (Fig. 3b). In this subsection analysis, LVEF was similar between all subgroups (Fig. 3c), whereas only patients with amyloidosis exhibited a higher LV mass index and T1 values (Fig. 3d, f). MCF was lower in patients with amyloidosis compared with athletes and HHD but similar to HCM (Fig. 3e). Atypical LGE score differentiated between amyloidosis and all other hypertrophy forms, between athletes and HHD and between athletes and HCM. However, LGE score did not differentiate between athletes and HHD (Fig. 3g). %normal myocardium differentiated between all groups with mild-moderate LVH, except between HCM and amyloidosis (Fig. 3h).

**Fig. 4:**
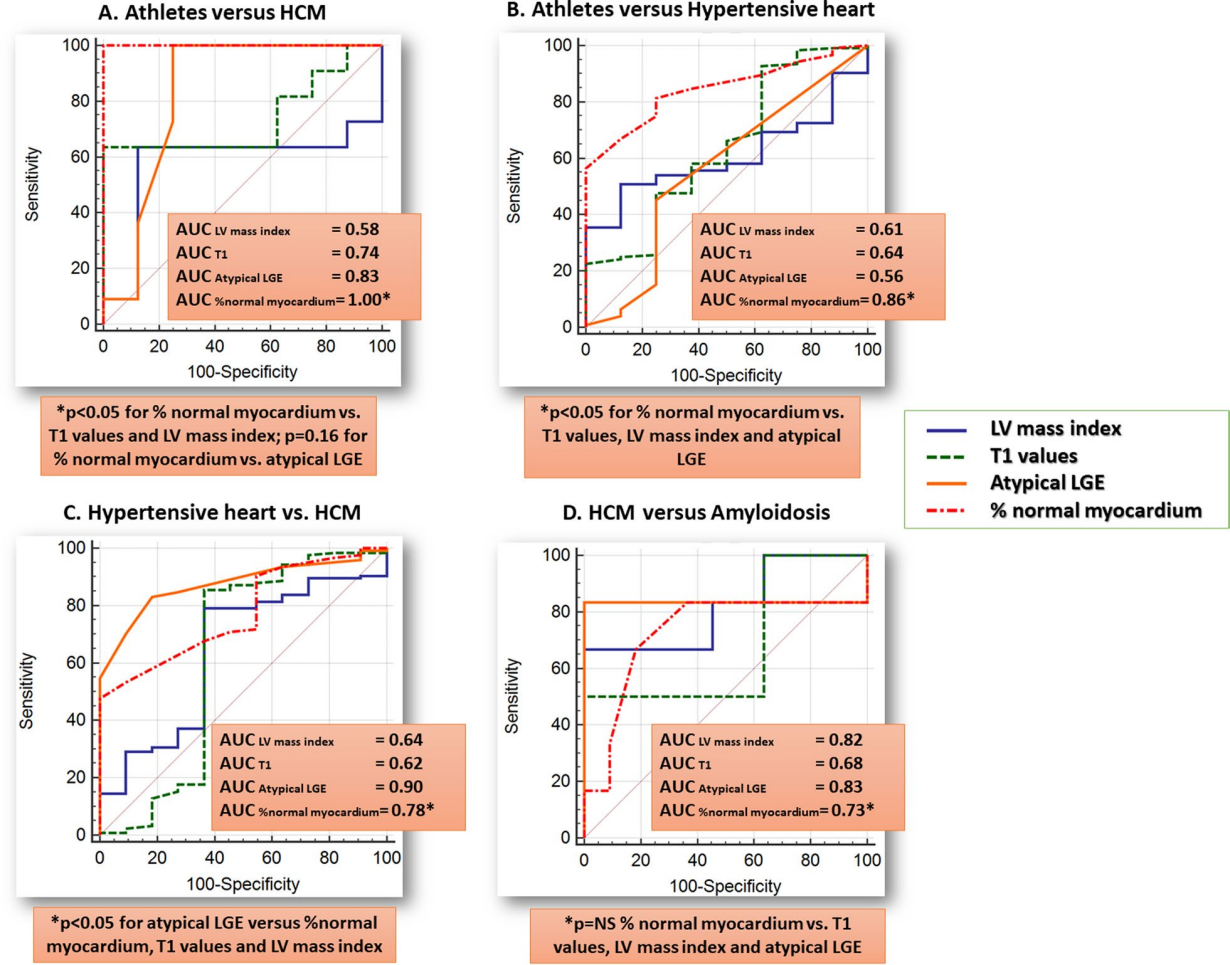
% normal myocardium exhibited excellent precision for the differentiation between athletes’ heart and HCM (**A**) and the highest precision for the differentiation between athletes’ heart and HHD (**B**). For the differentiation between HHD and HCM as well as between HCM and amyloidosis on the other hand, atypical LGE exhibited higher precision (**C**, **D**), albeit without reaching statistical significance vs. that provided by %normal myocardium

%normal myocardium exhibited excellent accuracy for the differentiation between athletes vs. HCM (AUC = 1.0, 95% CI = 0.85–1.0). For the differentiation between athletes and HHD, %normal myocardium exhibited higher accuracy than atypical LGE (∆AUC = 0.3, p = 0.003). For the differentiation between HHD and HCM atypical LGE exhibited wither accuracy than %normal myocardium (∆AUC = 0.12, p = 0.04), whereas a trend was noted for wither accuracy of atypical LGE compared to %normal myocardium for the differentiation between HCM and cardiac amyloidosis, without reaching statistical significance (∆AUC = 0.10, p = 0.11), (Fig. 4). Generally, both %normal myocardium and LGE score showed higher accuracies than T1 and LV mass index.

Overall higher accuracies could be obtained using the combination of %normal myocardium and LGE data (Table 2A, B). Diagnostic values remained similar after applying cut-off values from cohort A to cohort B (Table 2C). %normal myocardium provided excellent precision for the differentiation between athletes and HCM and significantly higher accuracy than LGE for the differentiation between athletes and HHD.

**Table 2:** Sensitivities, specificities, and accuracy values for the differentiation between different clinical entities by %normal myocardium, LGE data and by combining both

Clinical entities	Parameters	Sensitivity	Specificity	AUC	p-values
A. All patients					
Athletes vs. HCM	%normal myocardium	98%	100%	0.99	0.08^§^
Athletes vs. HCM	Atypical LGE	98%	83%	0.90	
Athletes vs. HHD	%normal myocardium	67%	91%	0.84	0.001^§^
Athletes vs. HHD	Atypical LGE	42%	83%	0.61	
HHD vs. HCM	%normal myocardium	47%	98%	0.78	0.03^§^
	Atypical LGE	74%	91%	0.88	
	%normal myocardium and LGE*	82%	98%	0.92	
HCM vs. amyloidosis	%normal myocardium	56%	91%	0.82	0.7^§^
	Atypical LGE	70%	96%	0.81	
	%normal myocardium and LGE**	80%	100%	0.94	
B. Patients with mild to moderate hypertrophy (IVS 11–15 mm)					
Athletes vs. HCM	%normal myocardium	100%	100%	1.0	0.16^§^
Athletes vs. HCM	Atypical LGE	100%	75%	0.84	
Athletes vs. HHD	%normal myocardium	83%	75%	0.86	0.003^§^
Athletes vs. HHD	Atypical LGE	41%	75%	0.56	
HHD vs. HCM	%normal myocardium	45%	100%	0.78	0.04^§^
	Atypical LGE	86%	85%	0.90	
	%normal myocardium and LGE*	82%	100%	0.92	
HCM vs. amyloidosis	%normal myocardium	85%	50%	0.73	0.11^§^
	Atypical LGE	83%	100%	0.83	
	%normal myocardium and LGE**	83%	100%	0.83	
*C. All patients (Cut-off values derived from cohort A and applied to cohort B; mean AUC values of cohorts A&B are provided)*					
Athletes vs. HCM	%normal myocardium	100%	100%	0.99	0.10^§^
Athletes vs. HCM	Atypical LGE	100%	67%	0.81	
Athletes vs. HHD	%normal myocardium	73%	91%	0.84	0.008^§^
Athletes vs. HHD	Atypical LGE	48%	67%	0.55	
HHD vs. HCM	%normal myocardium	39%	100%	0.78	0.42^§^
	Atypical LGE	64%	91%	0.87	
HCM vs. amyloidosis	%normal myocardium	56%	91%	0.83	0.38^§^
	Atypical LGE	77%	68%	0.82	

*AUC* area under the curve, *HCM* hypertrophic cardiomyopathy, *HHD* hypertensive heart disease, *LGE* late gadolinium enhancement, *IVS* intraventricular septum

*For the combined approach, patients with no atypical LGE were classified as HHD, whereas in patients with one or more segments with atypical LGE classification was performed by %normal myocardium

**For the combined approach, patients with ≥ 10 segments atypical LGE were classified as cardiac amyloidosis, whereas in patients with < 10 segments atypical LGE, classification was performed by %normal myocardium

^§^For comparison of atypical LGE vs. %normal myocardium

In addition, calculation of a base-to-apex segmental strain gradient further helped differentiating between HCM and amyloidosis with acceptable sensitivity and specificity (Additional file 2: Figure S2A, B).

### Multiparametric approach

**Fig. 5:**
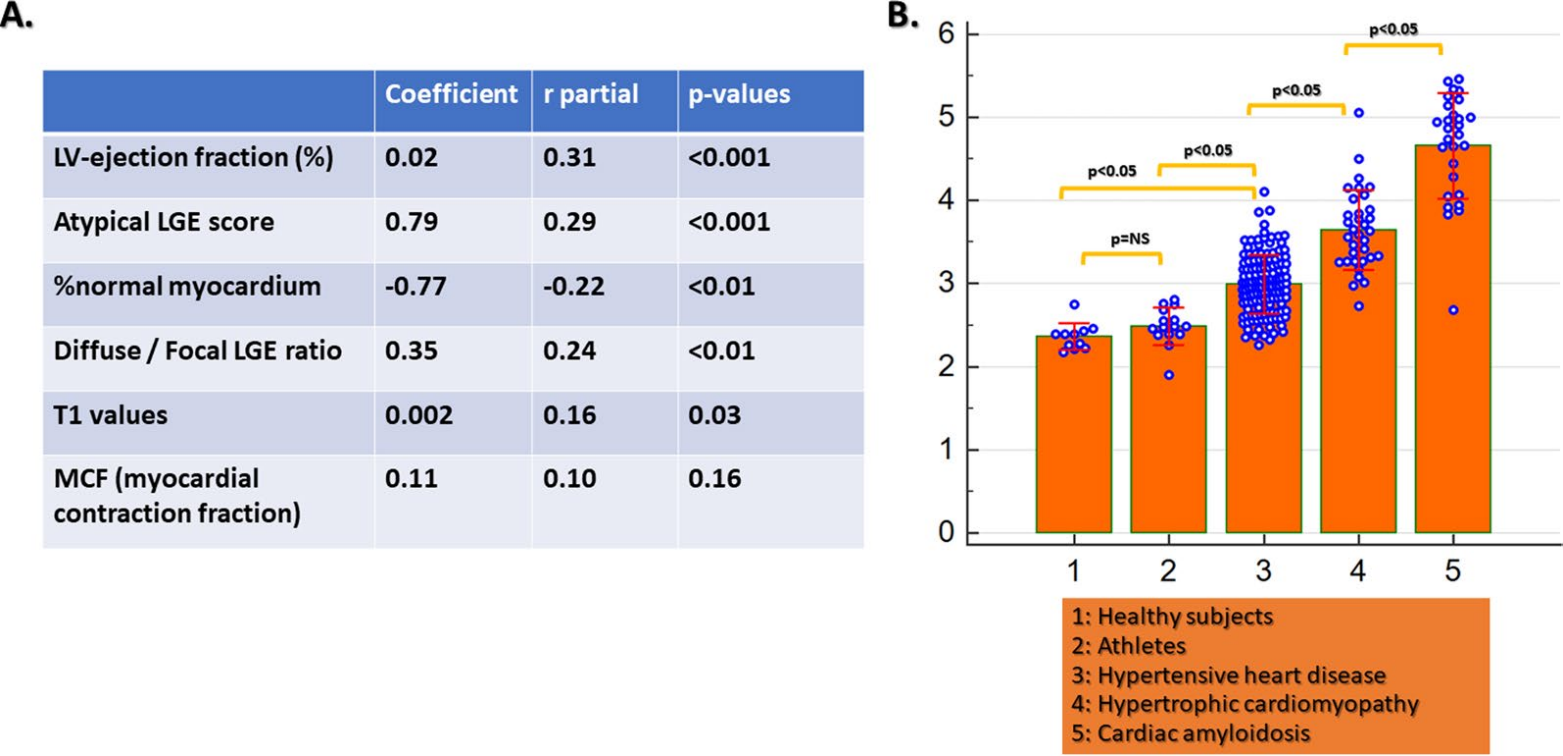
The combination of LVEF, LGE score and patterns, T1, MCF and %normal myocardium allowed for the precise differentiation between underlying pathologies (**A**, **B**)

Using a clustered based multivariable approach, the combination of LVEF, LGE score and patterns, T1, MCF and %normal myocardium allowed for the precise differentiation between underlying pathologies (Fig. 5a, b)

### Intra and interobserver variabilities

Intra- and interobserver variabilities for global strain were 1.3% and 1.5% for GLS, 1.8% and 2.2% for GCS and 3.9% and 4.5% for the assessment of %normal myocardium, respectively.

## Discussion

In this analysis of over 300 patients, we found that:

I. %normal myocardium can reproducibly assess myocardial strain but are only modestly related to LVEF and to LV mass index, T1 and LGE.
II. %normal myocardium, LV mass index, septal WT, T1 and LGE all differ to a certain degree between different clinical entities.
III. In patients with mild-moderate forms of LVH (septal WT between 11 and 15 mm), % normal myocardium offers excellent accuracy for the differentiation of athletes vs. HCM and of athletes vs. HHD.
IV. Combining atypical LGE score and patterns and % normal myocardium offers high precision for the differentiation between all studied underlying pathologies by a clustered multivariable approach. Specifically, good accuracies are provided for the differentiation between HCM vs. HHD and HCM vs. amyloidosis.

### Previous studies related to the differential diagnosis of LVH

Increase in LV WT is common in clinical practice and can be caused by either a change in the loading conditions, namely increased afterload, or through alterations of the myocardial structure itself, secondary to genetic defects (HCM) or infiltrative diseases (cardiac amyloidosis). Echocardiography is usually the first imaging tool used in patients with suspected LVH. It can readily identify other causes of LVH (i.e., aortic stenosis) and provides assessment of LV mass and LVEF. However, the latter has little use in differentiating LVH, as most forms of LVH show preserved LVEF until very late stages of the diseases [2]. This was also the case in our study cohort, where only patients with amyloidosis displayed reduced LVEF.

The athlete’s heart is characterised by a combination of LV dilatation and mild LVH [21]. However, the levels of septal WT can overlap with those seen in patients with early stages of HHD or HCM and mild to moderate LVH [22]. Furthermore, although the LVH is considered physiologic, several studies have pointed out to the presence of LGE in athletes. The pattern is atypical, i.e. non-ischemic, and its clinical relevance remains to be elucidated [23]. Indeed, in our study cohort, athletes had the most dilated LV and exhibited a mild LVH. Like in previous reports, 3 athletes also exhibited LGE in our study, all of them with an atypical pattern. With HCM on the other hand, approximately two thirds of the patients show LGE [24]. Several studies have looked at possible parameters extracted from conventional CMR acquisitions for differentiating between HCM and athlete’s hearts [25, 26]. However, for athletes who fall in the grey area of WT (13–15 mm), usually an interruption of sport is recommended, and longitudinal controls are performed to test for hypertrophy regression [27]. In our study cohort, when looking at the data for patients with overlapping LV WT, conventional parameters failed to establish a clear-cut difference between athletes and HCM. More recent studies focused on tissue characterisation (T1 mapping) for a better description of athlete’s heart. Similar to our study, they found a small decrease in T1 values in athletes in comparison to controls, which may mirror a decrease in extracellular volume and an increase in cellular mass [28]. Strictly adhering to the definition of LV WT > 15 mm for diagnosing HCM makes it very difficult to establish a differential diagnosis with HHD [29]. This is especially important in elderly patients, in whom arterial hypertension is very often diagnosed. Previous studies have pointed to LV mass index and more extensive LGE as better discriminators between the two conditions [29]. Lastly, the assessment of T1 values in myocardium was shown to provide some discriminatory power between patients with HCM and HHD [30]. Our findings correspond to these previous data. Amyloidosis on the other hand is another diagnosis, which needs to be considered in case of severe LVH [1]. Indeed, in our patients there was no difference in septal wall thickness between cardiac amyloidosis and HCM. Furthermore, LGE plays a significant role in differentiating between the pathologies. Thus, patients with amyloidosis exhibit a more broadly distributed LGE and higher T1 values [31, 31]. These findings were confirmed in our study.

### The added value of fast-SENC

SENC was developed in 2001 [33]. Since then, through improvements, the method allows the acquisition in a single heart-beat fast-SENC. SENC proved as a valuable tool for the identification of myocardial ischemia [15] and exhibits excellent reproducibility for the evaluation of global and segmental myocardial strain. We recently demonstrated the role of fast-SENC derived myocardial strain for the identification of all-comer patients with subclinical alterations of myocardial function, who later develop heart failure and in patients with non-ischemic cardiomyopathies, including dilated and HCM and cardiac amyloidosis [17, 34]. Fewer studies however, addressed the role of CMR derived myocardial strain for the characterization of LVH [35]. Similar to previous reports, we found no difference between athletes and heathy individuals in respect to %normal myocardium [36, 37]. This suggests that LVH associated with sport is a form of physiological hypertrophy and probably in most cases does not adversely affect function. In patients with HHD on the other hand, a decrease in %normal myocardium was noted in comparison to healthy individuals and athletes. Patients with HCM exhibited worse myocardial strain than those with HHD and even lower values were found in amyloidosis.

%normal myocardium offered excellent precision for the differentiation between the athletes’ heart and HCM and high accuracy for the differentiation between HHD and HCM, clearly surpassing the value of LVEF, mass index, T1 values and atypical LGE score. This is in line with our previous findings in patients with different stages of heart failure, where %normal myocardium identified all-comer patients with subclinical disease [15]. Interestingly, the correlations between strain and LVEF were not perfect. This is attributed to reduced strain values in patients with HHD, HCM and in some patients with amyloidosis and preserved LVEF. This is, however, an advantage of strain, because it helps to identify patients with normal LVEF but diminished strain, who have a much higher likelihood to convert to symptomatic heart failure in the future [17, 38]. In addition, it should be noted, that healthy subjects and athletes did not exhibit %normal myocardium = 100%, which may be misleading. However, this is attributed to the definition of %normal myocardium, where segments with longitudinal or circumferential strain ≤ − 17% are considered as normal. Based on earlier studies, it is known that regional differences are present with longitudinal and circumferential strain values in healthy subjects, with low longitudinal strain in the mid anterior, mid anteroseptal and apical lateral segments and low circumferential strain in basal anteroseptal segments and in the apical cap [17]. Due to such differences in the regional distribution, healthy subjects and athletes do not always show %normal myocardium of 100% but between 80 and 90%.

Because the differentiation between athletes and HCM or between athletes and HHD is often clinically challenging, especially in mild to moderate hypertrophy forms, the high precision of %normal myocardium in this setting, bears promising clinical implications. Furthermore, in both these clinical scenarios (athletes vs. HCM and athletes vs. HHD), %normal myocardium performed significantly better than T1 mapping. Although both sequences do not require the administration of contrast agents, fast-SENC acquisitions can be performed during free breathing within < 1 s and thus do not require long breath holds over ~ 10 heart beats as most current T1 mapping sequences. It should be noted however, that extracellular volumes (ECV) were not available in our study, and may have been superior to native T1, especially for the differentiation between amyloidosis and other forms of hypertrophy. For the differentiation between HCM and HHD and between HCM and amyloidosis with mild-moderate LVH, on the other hand, combining atypical LGE and %normal myocardium offered high accuracy rates, which surpassed that provided by other conventional CMR variables, such as T1, LVEF and LV mass index. In addition, for the differentiation between HCM and amyloidosis, a base-to-apex segmental strain gradient further aided the differentiation between the 2 clinical entities, in agreement with previous reports [18]. The distribution of LGE patterns on the other hand, was also different among different entities with focal LGE being predominantly present in athletes and diffuse LGE being increasingly present in HHD and HCM, whereas cardiac amyloidosis patients exhibited only diffuse LGE. Overall, the combination of atypical LGE score and %normal myocardium allowed for the precise differentiation of all underlying pathologies using a clustered multivariable approach. In addition, after diagnostic characteristics for atypical LGE score and %normal myocardium remained similar after applying cut-off values from a test cohort A to validation cohort B, which further demonstrates robustness of such a multiparametric approach.

## Limitations

Our cohort was extremely heterogenous in respect to demographic data. In addition, ECG data were not systematically analysed, whereas age and sex matching were not performed, which is a possible limitation due to possible variations of strain with age and gender [39]. Furthermore, not all forms of LVH were included in our analysis, especially patients with Anderson-Fabry disease and mitochondrial myopathies. Furthermore, overlapping phenotypes, for instance between HHD and HCM or HCM and amyloidosis, cannot be excluded. Indeed, a significant part of HCM and amyloidosis patients had arterial hypertension, whereas amyloidosis was recently reported to be diagnosed in a significant number of patients with initial diagnosis of HCM, especially in older patients [40]. We acknowledge this as a possible confounder of our study. However, all patients with HCM and amyloidosis exhibited controlled blood pressure values. In addition, different stages of each disease may provide different phenotypes with CMR, in terms of LGE or myocardial strain. However, a subsection differentiation of different disease stages for example in case of patients with amyloidosis was beyond the scope of our study. The definition of “normal values” when analysing strain values is still a matter of ongoing debate. Thus, the value of − 17% which we used from previous studies for fast-SENC cannot be extrapolated to other techniques used for the assessment of myocardial strain.

## Conclusions

Fast-SENC derived myocardial strain is a valuable tool for the characterisation of patients exhibiting LVH and can be used together with atypical LGE to help establishing an etiological diagnosis in forms of mild-moderate hypertrophy of unclear aetiology.

## Supplementary Information

**Additional file 1: Figure S1.** Poor correlations were observed between global longitudinal strain (GLS), global circumferential strain (GCS) and %normal myocardium with LVEF (A–C). Moderate correlations on the other hand, were depicted between %normal myocardium and LV mass (D), %normal myocardium and T1 values (E) and between %normal myocardium and atypical LGE score index (F).

**Additional file 2: Figure S2.** The base-to-apex segmental strain gradient was significantly higher in patients with amyloidosis vs. HCM and helped differentiating the 2 entities with acceptable sensitivity and specificity in patients with mild-to-moderate LVH (septal wall thickness ≤ 15 mm).

## Data Availability

The dataset used and/or analysed is available from the corresponding author upon reasonable request.

## Abbreviations

AUC: Area under the curve
BMI: Body mass index
BSA: Body surface area
CMR: Cardiovascular magnetic resonance
ECG: Electrocardiogram
ECV: Extracellular volume fraction
GCS: Global circumferential strain
GLS: Global longitudinal strain
HCM: Hypertrophic cardiomyopathy
HHD: Hypertensive heart disease
IVS: Interventricular septum
LGE: Late gadolinium enhancement
LV: Left ventricle/left ventricular
LVEDV: Left ventricular end-diastolic volume
LVEF: Left ventricular ejection fraction
LVESV: Left ventricular end-systolic volume
LVH: Left ventricular hypertrophy
MCF: Myocardial contraction fraction
MOLLI: Modified Look-Locker inversion recovery
RV: Right ventricle/right ventricular
SENC: Strain encoded sequence
WT: Wall thickness
