# Supplementary figures and images for: Multi-parametric assessment of left ventricular hypertrophy using late gadolinium enhancement, T1 mapping and strain-encoded cardiovascular magnetic resonance
Source: J Cardiovasc Magn Reson. 2021 Jul 12;23:92. doi: 10.1186/s12968-021-00775-8 (PMC8273957; doi:10.1186/s12968-021-00775-8)

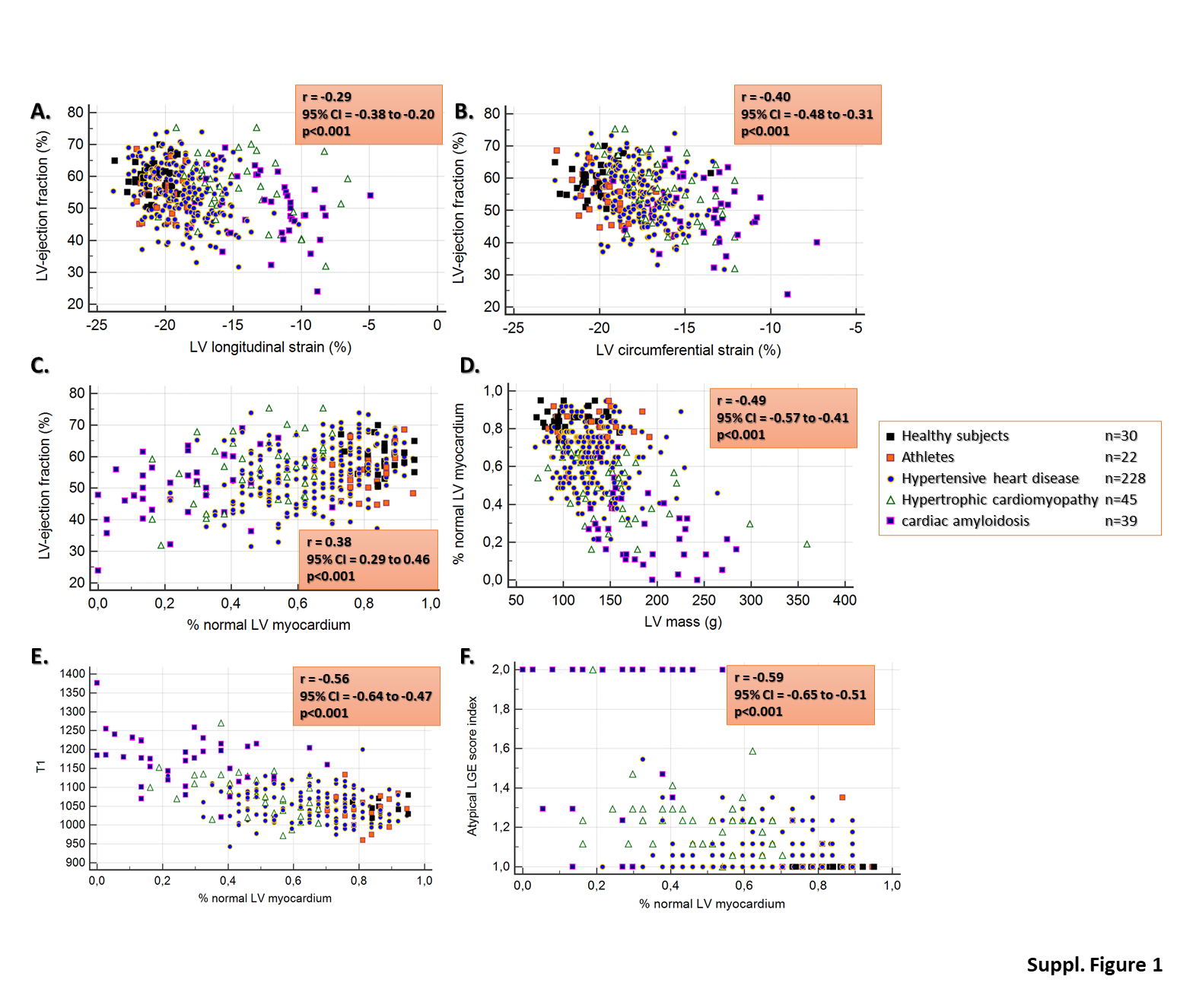

Supplement: Supplementary file 1 — Additional file 1: Figure S1. Poor correlations were observed between global longitudinal strain (GLS), global circumferential strain (GCS) and %normal myocardium with LVEF (A–C). Moderate correlations on the other hand, were depicted between %normal myocardium and LV mass (D), %normal myocardium and T1 values (E) and between %normal myocardium and atypical LGE score index (F). [file 12968_2021_775_MOESM1_ESM.tif]

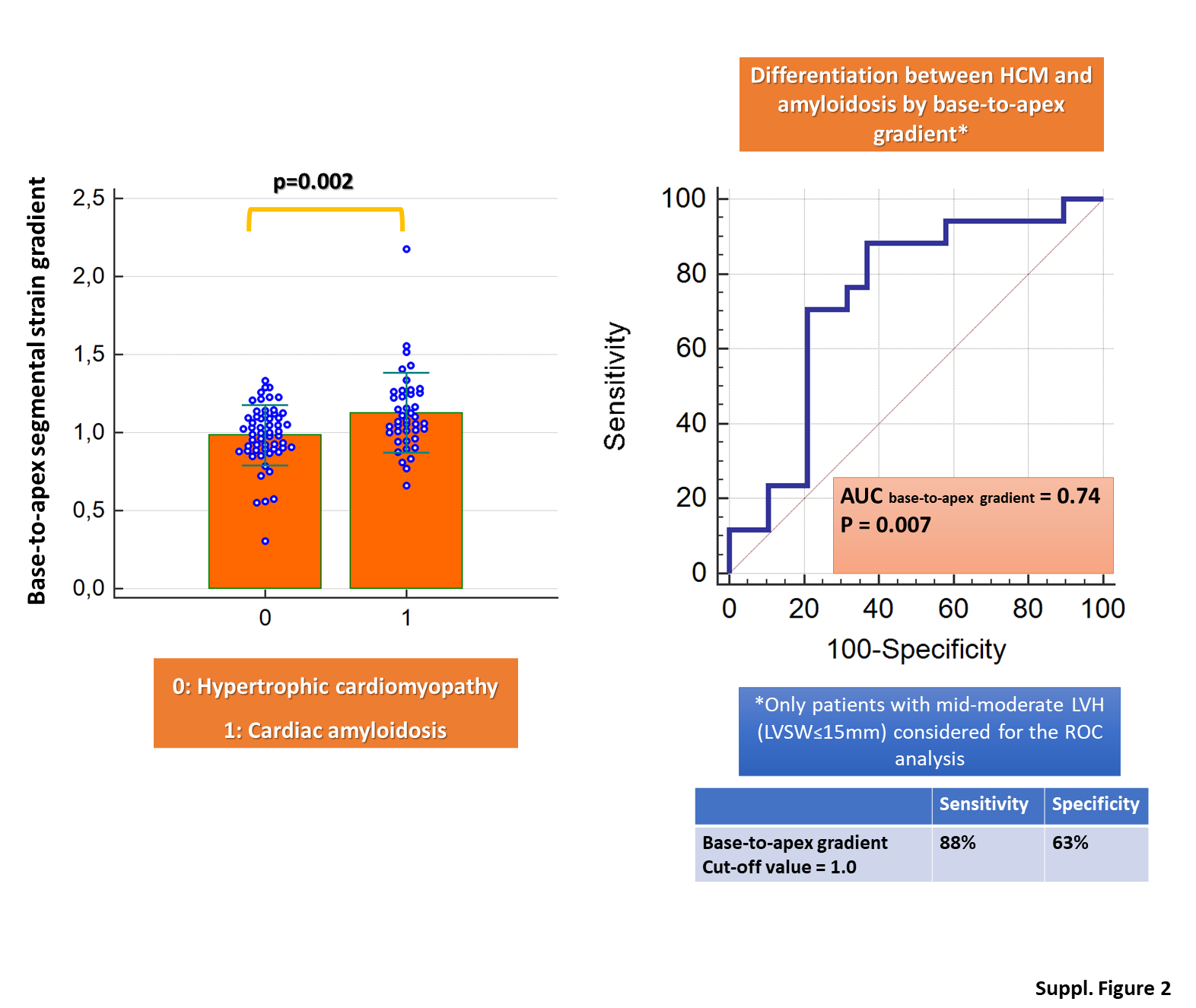

Supplement: Supplementary file 2 — Additional file 2: Figure S2. The base-to-apex segmental strain gradient was significantly higher in patients with amyloidosis vs. HCM and helped differentiating the 2 entities with acceptable sensitivity and specificity in patients with mild-to-moderate LVH (septal wall thickness ≤ 15 mm). [file 12968_2021_775_MOESM2_ESM.tif]
